# Potential effects of samsum ant, *Brachyponera* sennaarensis, venom on TNF-α/NF-κB mediated inflammation in CCL4-toxicity in vivo

**DOI:** 10.1186/s12944-016-0364-7

**Published:** 2016-11-18

**Authors:** Jameel Al-Tamimi, Ibrahim M. Alhazza, Mohamed Al-Khalifa, Ali Metwalli, Ahmed Rady, Hossam Ebaid

**Affiliations:** 1 Department of Zoology, College of Science, King Saud University, P.O. Box 2455, Riyadh, 11451 Saudi Arabia; 2 Department of Food Science, College of Agriculture and Food Science, King Saud University, Riyadh, Saudi Arabia

**Keywords:** CCL_4_, Samsum ant venom, Antioxidant activity, NF-κB and IκB, TNF-α

## Abstract

**Background:**

Ant venom shows antimicrobial, anti-parasitic and anti-inflammatory activities, both in vitro and in vivo. Our recent studies have confirmed the role of samsum ant venom (SAV) as a powerful antioxidant. This study aimed to investigate whether SAV as a potential treatment for CCl4-induced acute liver toxicity in an animal (rat) model.

**Methods:**

Thirty-two rats were assigned into four groups; the first one served as the control. The second group received a single dose of 1 ml/kg CCl_4_ in a 1:1 ratio with olive oil through an intraperitoneal injection. The third group received a single dose of 1 ml/kg CCl_4_ and then treated with SAV at a dose of 100 μg SAV twice a week for three weeks. The fourth group received a dose of 100 μg SAV only twice a week for three weeks. ELISA, RT-PCR and histopathological examinations were applied.

**Results:**

Results showed that antioxidant enzymes were significantly reduced in the diseased animals. SAV was found to significantly restore the oxidative stability in diseased animals. ELISA estimation and RT-PCR analysis also showed significant upregulation of both nuclear factor (κB) NF-κB and inhibitor (κB) IκB, respectively, in the diseased animals compared to the normal ones. The expression of tumour necrosis factor alpha (TNF-α) and pro-apoptotic receptor (Fas) were also significantly up-regulated in the diseased rats. Interestingly, SAV was found to significantly restore NF-κB, IκB and TNF-α in the diseased rats to the normal values. As a result, liver enzymes, serum proteins and lipid concentrations were significantly improved by SAV in CCl4-animals in comparison with the control ones. Moreover, SAV obviously improved the hepatic tissues of the same group was.

**Conclusion:**

SAV treatment restores the normal biochemical and oxidative stability by improving the TNF-α/NF-κB mediated inflammation in CCL4-treated rats.

## Background

Pharmaceutical scientists are constantly searching for molecules of therapeutic benefit. Venoms are a promising source for the discovery of active molecules as they offer potential biologically active properties [1], that may be useful as new tools for the design of drugs [2]. Venoms exhibit a potential activities including antimicrobial, haemolytic, cytolytic, paralytic and insecticidal pharmacologies. Ant venoms exhibit greater variability in composition and function than do venoms from any other arthropods [3].

Ant venom-toxic reactions are caused by substances, including acids and alkaloids [4]. The venom of the ant *Odontomachus bauri* has been demonstrated to show antimicrobial activity against bacteria, as well as anti-parasitic activity against *Toxoplasma gondii* infection [5]. The venom gland secretions of Samsum ant (SAV) *Pachycondyla species* contain volatile components [6].

Carbon tetrachloride (CCl_4_) is a well-known chemical compound causing hepatic injury [7]. It has been widely used to induce liver injury, fatty liver and liver fibrosis in experimental animals, and a single dose of CCl_4_ can lead to centrosomal necrosis and steatosis [8]. CCl_4_ is capable of reproducing hepatic cirrhosis via the metabolic changes resulting from free radicals [9]. Liver injury with CCl_4_ includes the production of free radicals and the activation of macrophages.

In this context, this study provides insights into the pharmacological applications of SAV by estimating the inducible transcription nuclear factor-κB (NF-κB) which is a central regulator of inflammatory and immune responses. The inhibitor of κB (IκB) is a well-defined regulator of NF-κB that resides in the cytoplasm and prevents NF-κB from nuclear entry by sequestration.

## Methods

### Collection of the samsum ant dissection of the venom gland

Collected ant’s Samsum colonies from Hotat Bani Tamim Governorate, East Riyadh, Kingdom of Saudi Arabia. Extracted dirt block containing the nest ants and put in a cloth bags were moved to the ant insectary in the Zoology Department, King Saud University. In addition, placed in plastic containers capacity of 20 × 70 cm, painted upper interior edges with grease to prevent the exit of ants, and provided a glass tube with sugar solution 10%, food added every day and consists of wheat grains crushed or pills tile of the nest spray volumes of water twice a day according. The insect is grabbing in the area pregnant with forceps, grab the other machine stinging other forceps, and then quietly removed the separation of the venom gland. The venom glands were homogenized and then centrifuged at 1000 rpm for 2 min. and the supernatant was collected in tube Eppendorf stored at −25 °C in PBS buffer until use.

### Experimental design

Thirty-two Wister albino rats of male sex weighing 220–270 g (20 ± 1 weeks) were obtained from the faculty of pharmacy, king Saud University, Saudi Arabia. The animals were acclimated to the laboratory conditions two weeks before the start of experiment animals were then housed in stainless steel wire cages (4 animals/cage), under pathogen-free conditions. Animals were maintained were provided food and water. The temperature was controlled at (22 ± 2 °C) with a relative humidity of (45–65%) and lighting on a light/dark cycle of 14/10 h. Animals were weighed before dosing and at appropriate times and observed every second day to for signs of ill health. The study protocol was approved by the Animal Ethics Committee of the Zoology Department in the College of Science at King Saud University. Rats were assigned into four groups; the first one served as the control. The second group received a single dose of 1 ml/kg CCl_4_ in a 1:1 ratio with olive oil through an intraperitoneal injection. The third group received a single dose of 1 ml/kg CCl_4_ and then treated with SAV in low dose of 100 μg/Kg body weight on five occasions with two day intervals. The fourth group received a dose of 100 μg SAV on five occasions with two day intervals.

### Sample collection

Animals were sacrificed by ether anesthesia after 24 h. of the last SAV dose. Blood was collected by two separate tubes, in tubes with EDTA, and centrifuged at 3000 rpm for 10 min. The sera was separated and stored at −25 °C. The liver is gently excised and rinsed with BPS (7.4) to eliminate blood contamination, dried by blotting with filter paper was kept BPS. The tissue was then kept in freezer directly at −80 °C until analysis of antioxidant and oxidative stress.

### Homogenate preparation

The liver was perfused with (PBS) and homogenized, and the obtained samples were centrifuged at 10000 × g for 15 min at 4 °C. The supernatant was sucked and kept on ice until used to assay GSH, CAT and MDA activity.

### Blood biochemical parameters assay

Blood biochemistry parameters of the study were estimated and included cholesterol, triglyceride, total protein (TP) and albumin. In addition, some of blood enzymes such as alanine aminotransferase (ALT), aspartate aminotransferase (AST), alkaline phosphatase (ALP) all plasma biochemical parameters analyzed using reagent kits (Quimica Clinica Aplicada S.A., Spain), by Spectrophotometric analyzer, Pharmacia Biotech, (Ultrospec 2000, UV- Visible) Cambridge, England.

### Estimation of catalase (CAT) and GSH bioactivity

The activity of an antioxidant enzyme is assayed with standard protocol for catalase (CAT) was done by decomposition of hydrogen peroxide [11]. A glutathione (GSH) assay was carried out as previously mentioned [12]. Briefly, A 10% (w/v) homogenate of each frozen tissue was prepared. The resulting supernatant was boiled to deactivate and precipitate other proteins. GSH concentrations were then measured by adding 100 μl of boiled supernatant to 400 μl PBS. GSH concentrations were then determined by measuring the absorbance (OD) of the reaction after 1 min at 340 nm using a UV Visible Spectrometer (Ultrospec 2000, Pharmacia Biotech). GSH standards were measured concurrently to obtain a standard curve that was used to calculate GSH concentrations in samples. Results were expressed as μg GSH/g tissue.

### Determination of lipid peroxidation

Lipid peroxidation in homogenates was estimated spectrophotometrically following the method described by Okhawa et al*.* [13, 14]. An aliquot of 0.5 ml of the resulting supernatant was shaken with 2.5 ml of 20% trichloroacetic acid. To the resulting mixture, 1 ml of 0.67% thiobarbituric acid was added, shaken, and warmed for 30 min in a boiling water bath, and followed immediately by rapid cooling in ice for 5 min. 4 ml of n-butyl-alcohol was added and the sample was shaken well. The resulting mixture was then centrifuged at 16,000 × g for 5 min. The resultant n-butyl-alcohol layer was transferred to a separate tube and MDA content was determined spectrophotometrically at 535 nm using a UV Visible Spectrometer (Ultrospec 2000, Pharmacia Biotech).

### ELISA estimation of FAS, NF-κB and TNF-α

The liver homogenate was tested for the protein level of FAS, NF-κB and TNF-α by ELISA according to the manufacturer’s instructions for the corresponding rat immunoassay kits (MyBioSource and Ray biotic, US). The optical densities were measured at 450 nm. The detection limits were set according to the log-log correlative coefficient of the standard curve.

### RT-PCR analysis of RNA expression of FAS and IκB

Quantification of mRNA expression by real-time polymerase chain reaction cDNA from the above preparation was subjected to PCR amplification using 96-well optical reaction plates in the ABI Prism 7500 System (Applied Biosystems®). The 25-μl reaction mixture contained 0.1 μl of 10 μM forward primer and 0.1 μl of 10 μM reverse primer (40 μM final concentration of each primer; chosen from pubmed com), 12.5 μl of SYBR Green Universal astermix, 11.05 μl of nuclease free water, and 1.25 μl of cDNA sample. The RT-PCR data was analyzed using the relative gene expression method, as described in Applied Biosystems ® User Bulletin No. 2. The data are presented as the fold change in gene expression normalized to the endogenous reference gene and relative to a calibrator.

### Histological sections

Liver parts were collected from the sacrificed and immersed in neutral buffered formalin 10% for at least 24 h. Tissues were fixed in Bouin’s fixative, processed into paraffin, cut into 4 μm thick sections by a rotary microtome were prepared (by Automatic tissue processor, Auto technique). Sections were stained with Haematoxylin and Eosin (H&E) for general histological architecture. The light microscopic images of rat liver slices of control, CCl_4_, CCl_4_ + 100 μg/kg SAV and 100 mg SAV were shown in Figure. Sections were stained with Haematoxylin and Eosin (H&E) for general histological architecture.

### Statistics

Data was analyzed using statistical package for social sciences (SPSS) version 20.0. determined the statistical significance of the differences between the averages of the results of the various groups were the use of analysis of variance unidirectional measurements repeated One Way ANOVA -Post Hoc Tukey’s test method for pairwise comparisons. This was in the form of data representation average ± standard error was adopted abstract level (*P* <0.05).

## Results

### Effect of samsum ant venom on oxidative stress

**Fig. 1:**
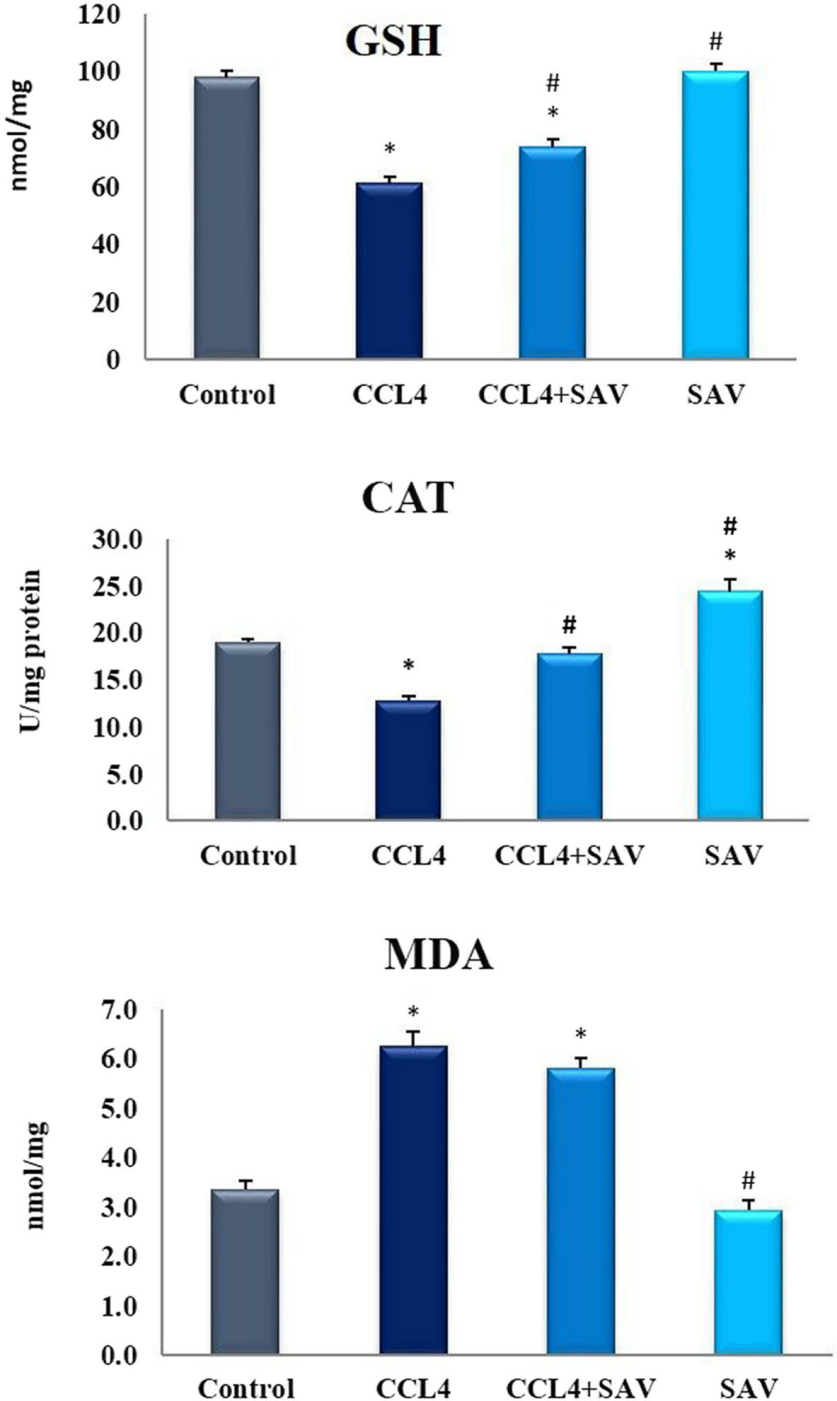
Estimation of catalase (CAT), glutathione (GSH) and malondialdehyde (MDA) changes in the liver of rats challenged with CCL4 challenge and treated with SAV. Values are expressed as the means ± standard errors. * shows statistically significant differences at *P* < 0.05 from the control. # shows statistically significant differences at *P* < 0.05 between any group compared to the CCL4 group

The level of catalase (CAT), reduced glutathione (GSH), and malondialdehyde (MDA) were taken as major parameters to assess the antioxidant status in the major organs of the liver of the treated animals. Samples in the CCL_4_ group showed a significant decrease (*p* < 0.01) in the level of CAT and GSH levels by 33% and 37.5%, respectively, compared with the control group. The CCL_4_ + SAV group, however, showed a significant increase in these levels compared to the CCL_4_ group (Fig. 1). The CCl_4_ treated group showed significantly increased (*p* < 0.01) levels of MDA (by 86.83% of MDA in liver samples compared to their respective controls. The CCL_4_ + SAV group, however, showed a significant decrease by 7.6% compared to the CCL_4_ group (Fig. 1).

### Effect of SAV on NF-kB and IkB

**Fig. 2:**
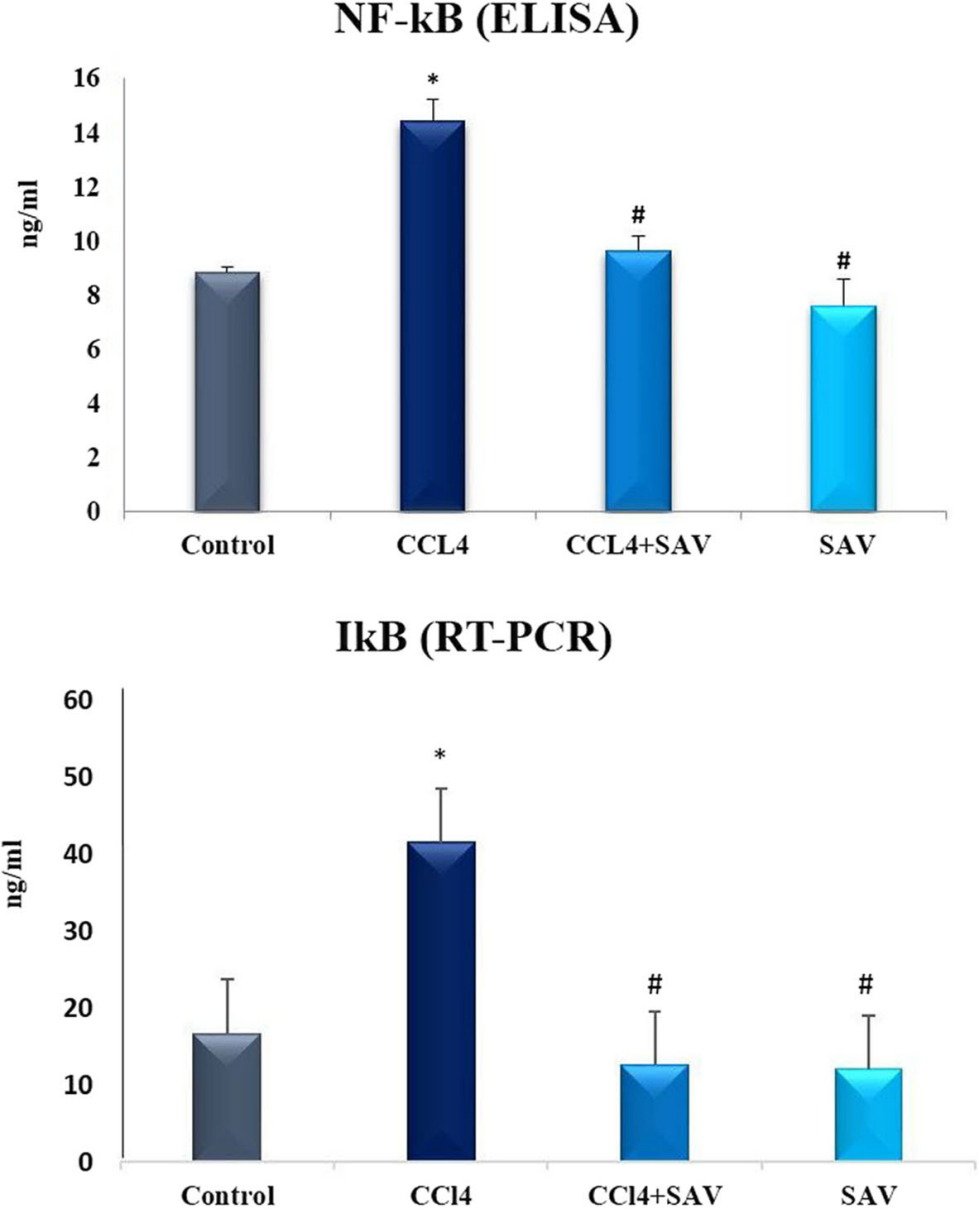
Estimation of the protein level of inducible transcription nuclear factor-κB (NF-κB) by ELISA and the RNA expression of the inhibitor of κB (IκB) by RT-PCR

We estimated NF-κB, which is a central regulator of inflammatory and immune responses. IκB is a well-defined regulator of NF-κB that resides in the cytoplasm and prevents NF-κB from nuclear entry by sequestration. Results showed that the protein level of NF-κB and the RNA expression of IκB are each significantly elevated (*p* < 0.01) in the CCl_4_ group compared to the control group. Interestingly, SAV was found to significantly restore the protein level of NF-κB and the RNA expression of IκB to normal levels, as shown in Fig. 2.

### Effect of SAV on TNF-α

**Fig. 3:**
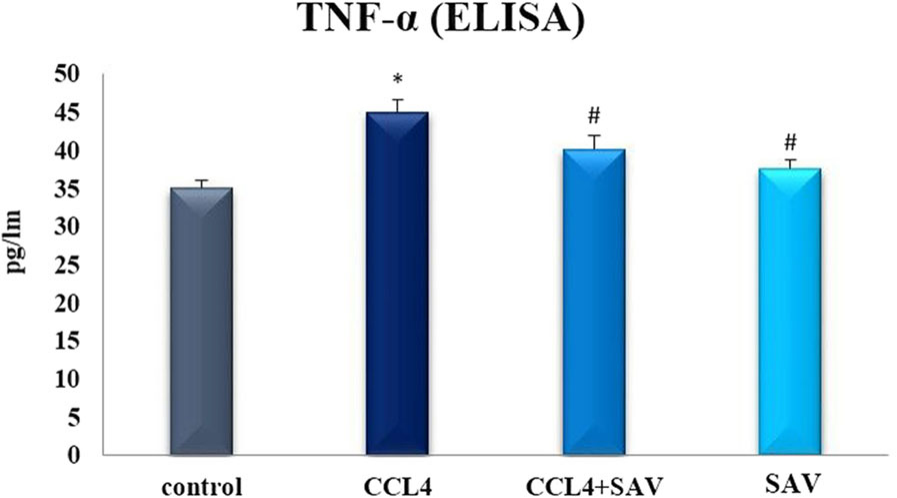
Changes in liver protein levels of TNF-a following CCL4 challenge and treatment with SAV. Values are expressed as the means ± standard errors. * shows statistically significant differences at *P* < 0.05 from the control. # shows statistically significant differences at *P* < 0.05 between any group compared to the CCL4 group

Because TNF-α is one of the inflammatory cytokines which is controlled by NF-κB, it behaved in a similar way to that of NF-κB. Results showed that the protein level of TNF-α was significantly elevated in the CCl_4_ group compared to the control group. SAV, meanwhile, was found to significantly restore the protein level of TNF-α to the normal levels, as shown in Fig. 3.

### Effect of SAV on Fas

**Fig. 4:**
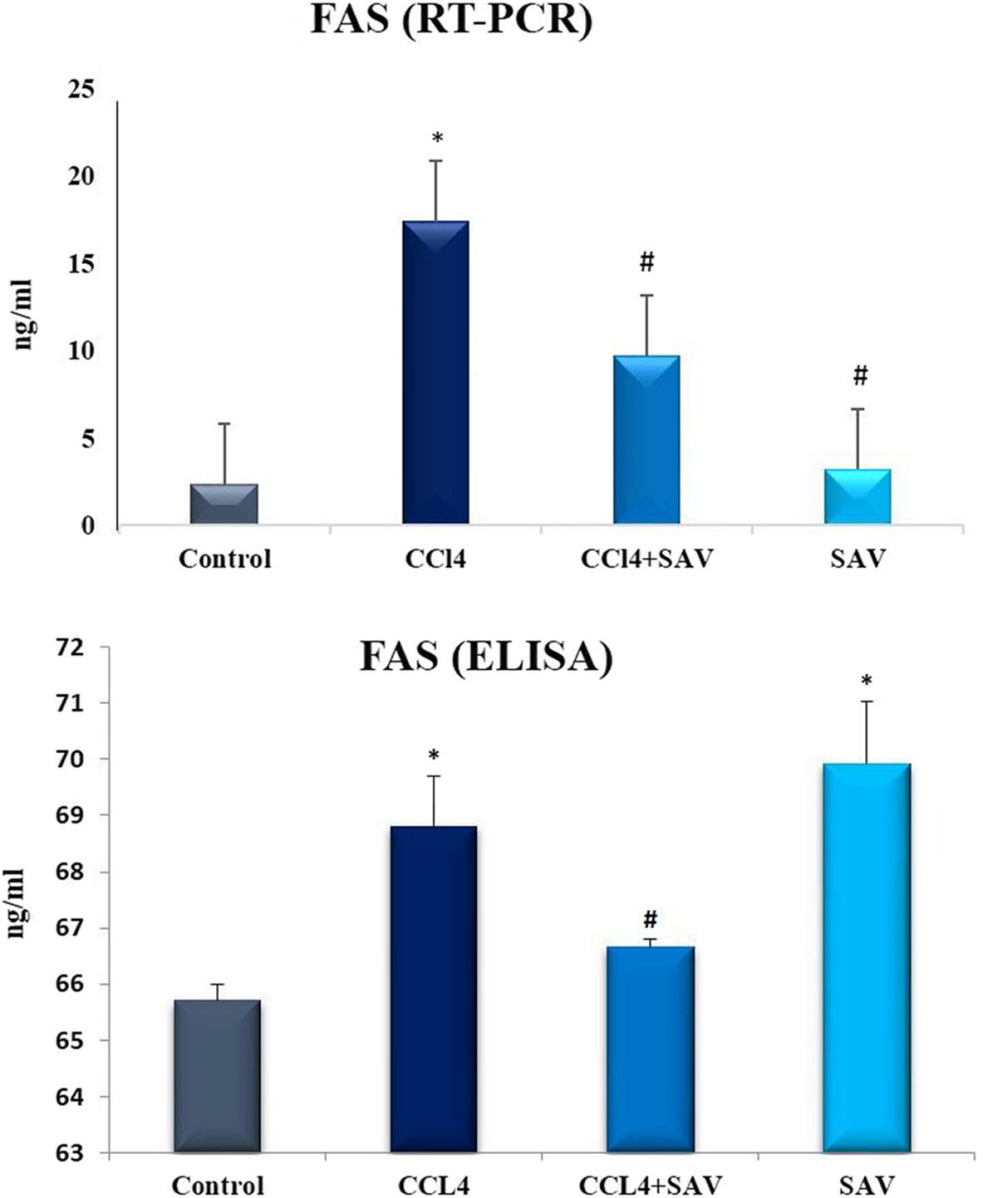
Changes in liver protein levels (ELISA) and the RNA expression (RT-PCR) of Fas following CCL4 challenge and treatment with SAV. Values are expressed as the means ± standard errors. * shows statistically significant differences at *P* < 0.05 from the control. # shows statistically significant differences at *P* < 0.05 between any group compared to the CCL4 group

Fas plays an important role in the regulation of immune response and apoptosis via inflammatory cytokines such as TNF-α. The Fas level in the CCl_4_ treated group showed a significant increase (*p* < 0.01) by 4.7% compared to the control group, while CCL_4_ + SAV group showed a decrease of 3% compared to CCL_4_ group and an increase of 1.5% compared to the control (Fig. 4).

### Histological sections

**Fig. 5:**
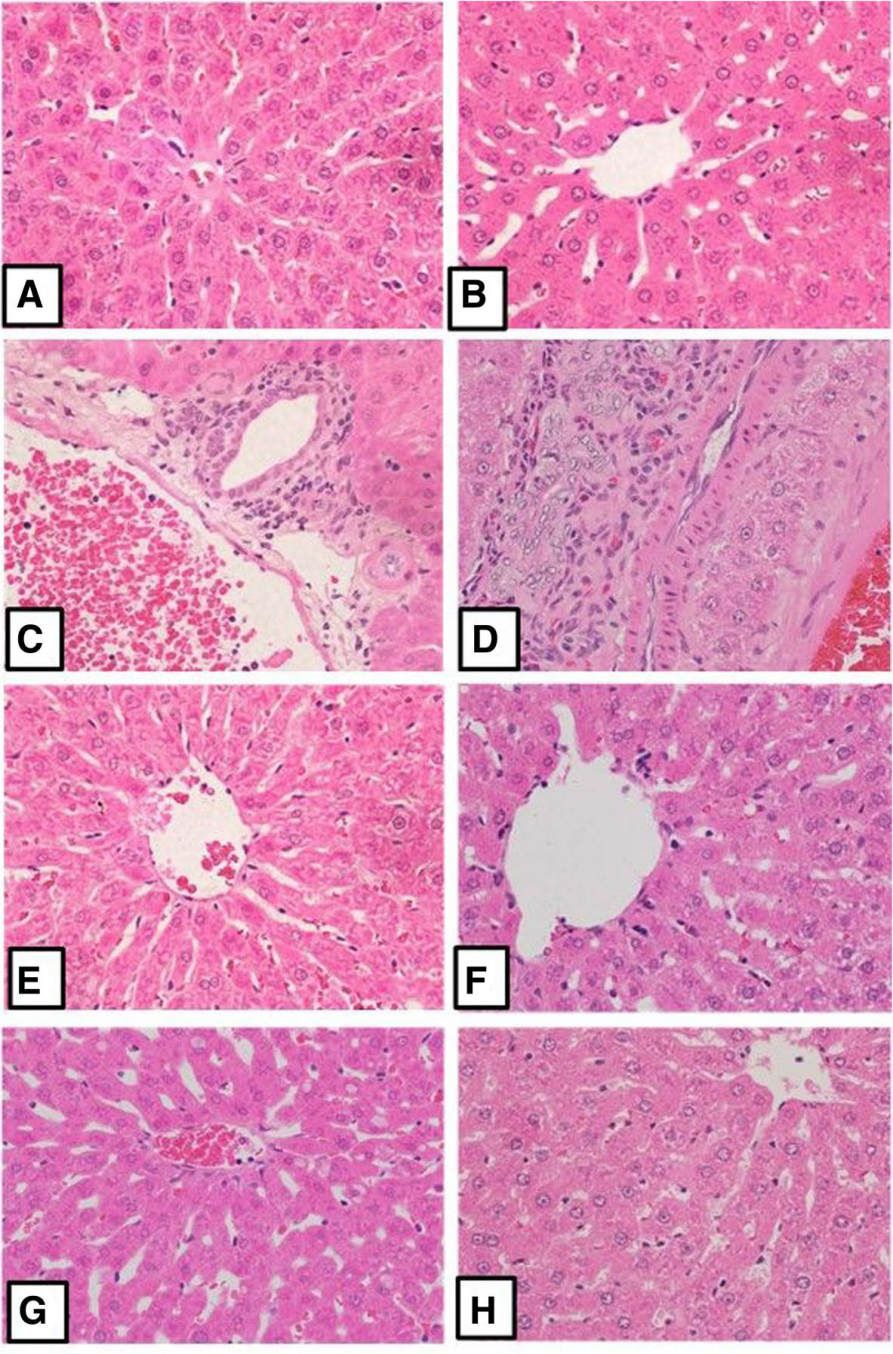
Representative histological features of the liver sections from control animal group (**a**, H&E, X200; **b** H&E, X400). A representative section from CCL4-treated group showing the increased vacuolation (**c**, **d**; H&E X400). SAV showed a remarkable improvement in the histological architecture of CCL4 + 100 SAV group (**e**, **f**; H&E X400). 100 SAV group (**g**, **h**; H&E X400)

The liver histology of the control group rats is shown in Table 1 and Fig. 5, where normal hepatocyte, central vein and histological architecture of hepatic tissues can be observed. The histological features of the CCl_4_-treated rats showed several visible histological changes to the liver, disruption of lobular architecture, including inflammatory cellular infiltrations, an increased number of mitotic figures, hepatic cell necrosis and thick vessel walls. This CCl_4_-induced damage to the liver was significantly alleviated in the CCL_4_ + SAV treated rats, with only minor side effects evident, such as faintly stained nuclei. Among the SAV treated rats, a general hepatic architecture with narrow hepatic sinusoids and a large number of hepatocytes is shown (Table 1, Fig. 5).

**Table 1:** Histological features of the liver sections from the control, CCL4-treated and CCL4 + SAV groups

	Disruption of lobular architecture	Inflammatory infiltrations	Mitotic figures	Hepatcytic necrosis	Dilated CV
Control rats	0	0	0	0	0
CCl_4_-treated rats	3	3	3	3	3
CCL_4_ + SAV rats	1	1	0	1	1

*CV* Central vein

### Effect of samsum ant venom on liver function markers

In the present study, levels of AST, ALT and ALP in plasma have been chosen as liver function markers. The CCl_4_ treated group showed that ALT and AST levels were significantly increased as compared to the control group. This concentration was significantly decreased in the CCL_4_ + SAV group, however (Table 2). On the other hand, no significant changes were observed in the concentration of ALP in any of the investigated rats.

**Table 2:** Liver functions, cholesterol, triglycerides, total protein and albumin levels in different rat groups

	Control	CCL4	CCL4 + SAV	SAV
ALT [IU/L]	42 ± 5.5	139 ± 9.3*	102 ± 8.7*#	60 ± 4.2#
ALP [IU/L]	180 ± 10.8	190 ± 9.7	185 ± 12.4	170 ± 10.3
AST [IU/L]	50 ± 3.2	165 ± 11.6*	110 ± 12.3*#	52 ± 6.3#
Cholesterol mg/dl	130 ± 12.5	255 ± 14.9	180 ± 11.1	135 ± 9.2
Triglycerids mg/dl	35 ± 5.2	75 ± 7.6	60 ± 5.6	30 ± 2.3
Total protein g/dl	6.5 ± 0.5	4.9 ± 0.2	5.5 ± 0.3	6.8 ± 0.4
Albumin g/dl	3.5 ± 0.1	3 ± 0.06	3.2 ± 0.12	3.3 ± 0.15

Values are means ± standard errors. * shows statistically significant differences at *P* < 0.05 from the control. # shows statistically significant differences at *P* < 0.05 between any group compared to the CCL4 group

### Effect of samsum ant venom on blood lipid concentration

Blood lipid concentration is a significant indicator of the ability of the liver to uptake different lipid derivatives. In the CCL_4_ only treated group, a significant up regulation of the cholesterol concentration in plasma was evident (by 94% as compared to the control). In the CCL_4_ + SAV group, however, this figure significantly decreased by 29% compared to the CCL_4_ treated group. In a similar way, the concentration of triglycerides in the CCL_4_ only treated group showed significant elevation (*p* < 0.01) by 111.4% compared to the control group, but this reading was significantly decreased by 29.8% in the CCL_4_ + SAV group compared to CCl_4_ treated group, although this was still 62.8% higher than the control concentration of triglycerides (Table 2).

### Effect of samsum ant venom on total protein and albumin

Total protein concentration in the CCl_4_ group showed a significant decrease by 24% compared to the control group. While the CCL_4_ + SAV group showed an increase by 16.6% compared to the CCL_4_ group. Similarly, albumin concentration in the CCl_4_ group treated showed a significant decrease by 23.33% compared to the control group, while SAV was found to restore the concentration of the albumin to the control level (Table 2).

### Effect of SAV and CCL_4_ on blood parameters

The CCL_4_ group showed a significant increase in the total number of white blood cells compared with the control group. SAV was found to improve the total blood cell count in the CCL_4_ rats. Furthermore, there was an obvious improvement in the differential white blood counts in the rats treated with SAV together with CCL_4_ (Table 3).

**Table 3:** Changes in blood parameters caused by CCL_4_ challenge and SAV treatment

Blood parameters	Control	CCL_4_	CCL_4_ + 100 μg SAV	100 μg SAV
RBC X10^6^/mm3	8.45 ± 0.11	7.75 ± 0.27	7.22 ± 0.27	7.20 ± 0.13
Hb mg/100 mg	16.10 ± 0.18	15.32 ± 0.26	14.8 ± 0.63	14.83 ± 0.23
WBC x10^3^/mm3	9.60 ± 1.71	16.36 ± 1.17*	11.88 ± 0.97*#	10.55 ± 1.37#
Neut. x10^3^/mm3	29.88 ± 1.25	32.72 ± 2.71	10.8 ± 0.17*#	9.55 ± 1.03*#
Lymph. x10^3^/mm3	65.63 ± 1.30	63.10 ± 2.97	80.6 ± 0.40*#	83.60 ± 1.23*#
Mono. x10^3^/mm3	4.50 ± 0.22	4.18 ± 0.33	8.6 ± 0.35*#	6.85 ± 0.87*#

*Hb* haemoglobin, *RBC* red blood cells, *WBC* white blood cell, *Neut* neutrophil, *Lymph* lymphocytes, *Mono* monocytes

Values are means ± standard errors. *shows statistically significant differences at *P* < 0.05 from the control. **#** shows statistically significant differences at *P* < 0.05 between any group compared to the CCL_4_ group

### Effect of SAV and CCL_4_ on the weight of the rats

**Fig. 6:**
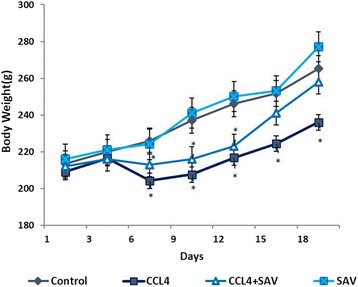
The average body weights of the rats in the different rat groups as measured every third day from the beginning until the end of the experiment until the end. * shows statistically significant differences at *P* < 0.05 from the control. # shows statistically significant differences at *P* < 0.05 between any group compared to the CCL4 group

The results showed (Fig. 6) the average body weight of the rats based on measurements taken every third day from the beginning of the experiment until the end. The results showed a significant decrease (*p* < 0.05) in the weight of rats in the CCL_4_ group, as well as, initially, in the CCL_4_ + SAV group compared to the control group. It was also observed that animals in the CCL_4−_group exhibited decreased appetite, diarrhoea, and a lack of activity. A significant improvement in the body weight after treatment with SAV was observed in the CCL_4_-treated rats (CCL_4_ + SAV), in particular during the last week of the experiment.

## Discussion

Recent studies have demonstrated that natural products and/or functional food-supplementation enhances the immune system, prevents DNA damage, and decreases the risk of a wide range of diseases [15–17]. Ant venoms are characterized by their high specificity and potency in acting on molecular targets and the immune system [18–21]. SAV was found to have the potential capability to restore oxidative stability after CCL_4_ acute nephrotoxicity [22, 23] in rats in a dose dependent manner [10]. The low dose of 100 μg/Kg body weight on three occasions with two day intervals resulted in the best outcomes from the point of view of the structure of the renal tissue, with a partial nut not complete recovery [10]. Thus, the low dose may be applied for a longer time in order to achieve a complete structural and functional recovery. It is important to report that low doses of SAV might help the rodents to adapt and optimize their physiological systems, as is evident from the histopathological evaluation in the present study. Thus, we used here SAV at a dose of 100 μg SAV five occasions with two day intervals. Many physiological indicators were measured, such as blood lipid profile, oxidative stress, antioxidant enzymes, glutathione, TNF-α, Fas and NF-κB.

Here, the imbalance between the production of ROS and the level of protection afforded by cellular antioxidants cause an oxidative stress [24]. The release of oxygen radicals causes tissue damage during inflammation [25]. We found that the level of MDA was elevated in diseased rats while, SAV restored the oxidative markers in these rats. Previous studies have shown that SAV plays an active role in scavenging free radicals [26]. Indeed, a significant enhancement of glutathione and catalase was observed in the SAV supplemented rats in this study.

NF-κB, which is stimulated by oxidative stress, is a central regulator of inflammatory and immune responses, while IκB is a well-defined regulator of NF-κB that resides in the cytoplasm and prevents NF-κB from nuclear entry by sequestration. NF-κB is required for the induction of pro-inflammatory cytokines, such as IL-1b, TNF-α and IL-6 [27]. We addressed the role of NF-κB in the present study. The oxidative stability induced by SAV appeared to mediate the activation of NF-κB, leading to a normalization in the regulation of the inflammatory cascade and the stimulation of tissue repair in SAV rats. Additionally, gene expression of pro-inflammatory cytokines, including TNF-α, and the protein levels of NF-κB, were significantly increased in tissues of the CCl_4_ group.

In the same way, SAV has a potential impact in stimulating the characteristic anti-inflammatory cytokines. SAV restored normal levels of TNF-α in diseased rats. The expression of the chemokines and adhesion molecules necessary for the recruitment of inflammatory cells to the site of injury is modulated by TNF-α [28, 29]. The extrinsic signalling cascade is activated by extracellular signals *via* cytokines, which include members of the TNF super family, *e.g*., TNF and Fas ligand, and their cell surface receptors. Fas is one of several important genes involved in the initiation or activation of apoptotic signalling pathways. Fas also plays an important role in the regulation of immune response. Here, the Fas protein was elevated in the diseased rats, which may indicate its role in the pathology of liver. Interestingly, gene expression of the pro-apoptotic receptor Fas was significantly up-regulated following CCL_4_ induction in rats. The elevated Fas level either indicates the increased susceptibility of CCl_4_ to apoptosis [30] or points to the pro-survival action of Fas itself. Thus, oxidative stress may induce cell apoptosis via Fas up-regulation and the Fas-mediated apoptotic pathway. Park et al. [31] found that deletion of Fas protects cells from the cytotoxic effects. In this context, it is interesting that the expression of Fas in hepatic tissue was significantly increased in CCL_4_ rats, while SAV treatment was found to restore the Fas level by a significant degree.

The results for the CCL_4_-injected rats revealed a clear decline in the number of neutrophils and organ functions, as has previously been found in [32, 33]. The CCL_4_-treated rats also showed an increase in the concentration of liver enzymes and a decrease in total protein and albumin. This may be due to releasing of many liver enzymes into the blood in the event of damage to the membranes of liver cells [34]. In contrast, in the CCL_4_+ SAV group, there was an evident improvement in both the blood profile and the liver function, indicating a positive role for SAV in tissue repair. The evidence of the restoration of liver function is particularly strikingly revealed in the improvement in albumin levels, which is one of the main goals of liver treatments. This improvement is due to the ability of SAV to reform the liver tissue through its ability to activate antioxidant enzymes and reduce oxidative stress in these tissues. These results are similar to those of Kim et al. [35] who proved the ability of bee venom to lower the concentration of liver enzymes after a significant increase in the serum of CCL_4_–injected mice.

Here, a single intraperitoneal dose of CCl_4_ caused severe hepatotoxicity in rats, as shown by the significant elevation of the serum lipid profile and the increased incidence of histopathological hepatic injury. This is consistent with previous studies that have showed that increased serum lipids is the most important mechanism by which CCL_4_ increases oxidation in cellular membranes [36, 37]. The damage in cell membranes may be due to increased ROS that affects cell metabolism and increases the oxidation of lipids, DNA and, protein, and this has been confirmed by numerous studies [32, 38]. We found that SAV was effective in rejuvenating the activity of antioxidant enzymes that had been lowered in the hepatic tissues of CCL_4−_animals. This has been attributed to the role of SAV inhibited free radicals. This is in accordance with a recent study that found that SAV had a hypolipidemic effect in rats after induced disruption of these parameters by LPS injection [39]. This is also consistent with the results reported in [40], which showed an improvement in the antioxidant levels in the serum of mice after injecting insect venom, as well as with other previous results [41, 42], which confirmed the ability of hornet venom to activate antioxidant enzymes, and the ability of bee venom to stimulate these same enzymes. There is an inverse correlation between blood lipid profile and T-cell proliferative capacity in dogs, where the reduction in total blood cholesterol, LDL and non-HDL-cholesterol levels was correlated with an increase in T-cell proliferation [43]. As it has a hypolipidemic effect, SAV therefore, can encourage the immune response.

## Conclusion

**Fig. 7:**
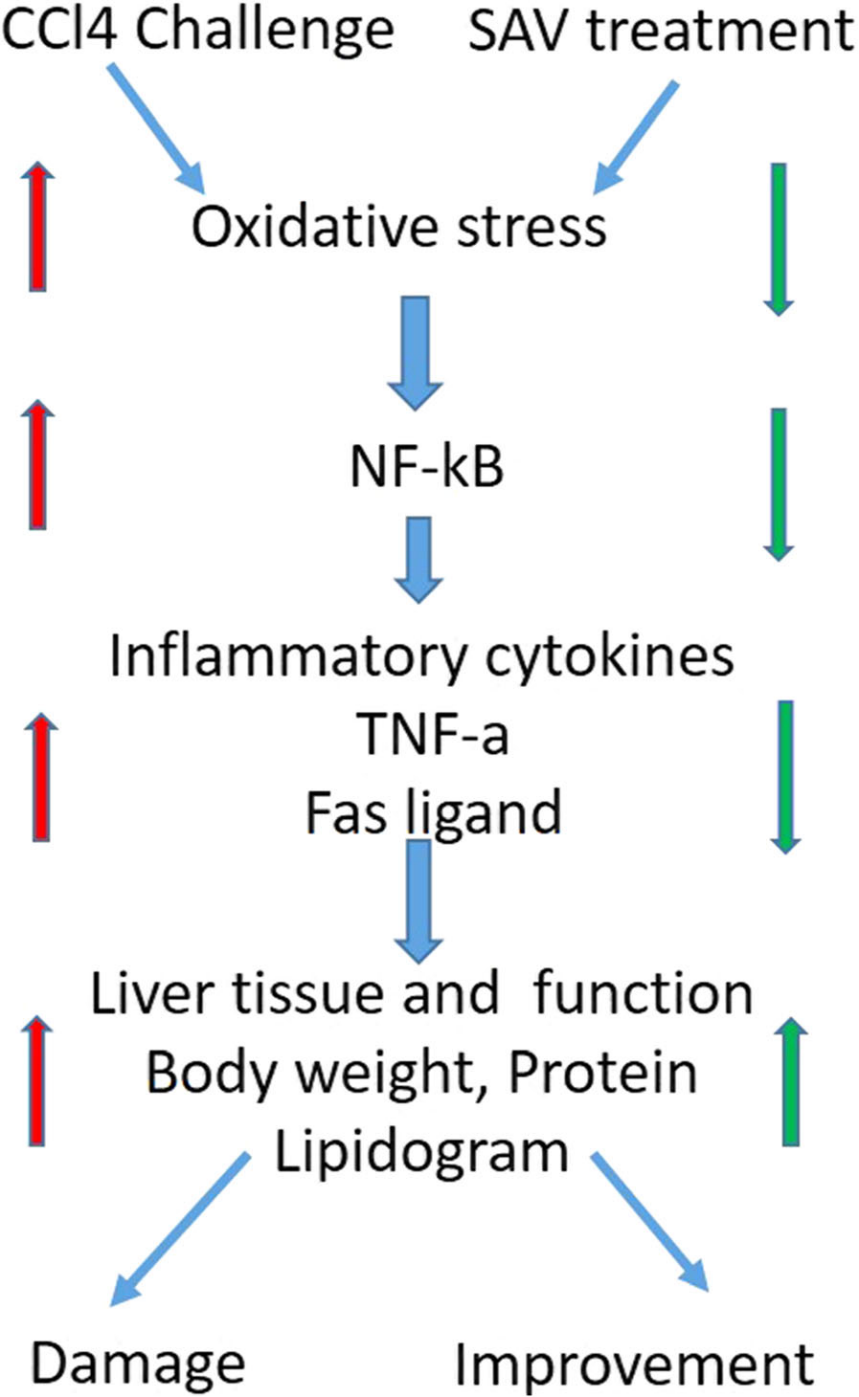
Diagrammatic summary of the relationship between CCl4 effects and SAV treatment of the rats

In CCl_4_ treated rats, oxidative stress causes tissue damage [23]. On the other hand, SAV greatly stimulated the normal inflammatory events of the healing process and then induced normal tissue formation. This result was confirmed by the improvement in the histological architecture of hepatic tissues as well as the normalization of liver functions. Taken together, the results of this work support the hypothesis that the oxidative stability and reduction in inflammation induced in CCl_4_ rats by SAV contributes to the accelerated restoration of tissue structure and functions (Fig. 7). In conclusion, SAV can effectively protect against CCl_4_-induced damage, and the mechanisms underlying this protective effect are potentially associated with improving the TNF-α/NF-κB mediated inflammation in CCL4-treated rats. The potential role of this natural product, SAV, needs to be intensively investigated in future research focusing on patient-oriented outcomes.

## Abbreviations

Akt1: Protein kinase B
CAT: Catalase
DTNB: 5,5-dithiobis-2-nitrobenzoic acid
EDTA: Ethylenediaminetetraacetic acid
FAS: Cell surface death receptor
GSH: Glutathione
MDA: Malondialdehyde
SAV: Samsum ant venom
TBA: Thiobarbituric acid
TCA: Trichloroacetic acid
